# Verification of junction dose between VMAT arcs of total body irradiation using a Sun Nuclear ArcCHECK phantom

**DOI:** 10.1002/acm2.12208

**Published:** 2017-10-29

**Authors:** Colm T. Morrison, Kirsty L. Symons, Simon J. Woodings, Michael J. House

**Affiliations:** ^1^ Department of Radiation Oncology Genesis Cancer Care WA Fiona Stanley Hospital 11 Robin Warren Drive Murdoch 6150 Western Australia; ^2^ School of Physics The University of Western Australia 35 Stirling Highway Crawley 6009 Western Australia

**Keywords:** ArcCHECK, junction dose, QA, VMAT TBI

## Abstract

A volumetric modulated arc therapy (VMAT) approach to total body irradiation (TBI) has recently been introduced at our institution. The planning target volume (PTV) is divided into separate sub‐volumes, each being treated with 2 arcs with their own isocentre. Pre‐treatment quality assurance of beams is performed on a Sun Nuclear ArcCHECK diode array. Measurement of junction regions between VMAT arcs with separate isocentres has previously been performed with point dose ionization chamber measurements, or with films. Translations of the ArcCHECK with respect to a known distance between the adjacent isocentres of two arcs, which are repeated with the ArcCHECK in an inverted position, allows the recording of a junction dose map. A 3%/3 mm global gamma analysis (10% threshold) pass rate for arc junctions were comparable to their component arcs. Dose maps of junction regions between adjacent arcs with different isocentres can be readily measured on a Sun Nuclear ArcCHECK diode array.

## 1 INTRODUCTION

Total body irradiation (TBI) is a conditioning regimen used for suitable patients with haematological malignancies receiving hematopoietic stem cell transplantation. In conjunction with chemotherapy, TBI kills malignant cells, and performs an immunosuppressive role to prevent immunologic rejection. Patients are conventionally treated on a linear accelerator (linac) at an extended source‐to‐surface distance (SSD) using static anterior‐posterior/posterior‐anterior (AP/PA) or parallel opposed beams. A nominal prescription of 12 Gy in six fractions is used at our institution. The dose is prescribed to the midline of the patient with the goal of delivering a homogenous dose, assisted by tissue compensators or shielding blocks to boost or limit dose to required areas. The doses from these techniques eradicate malignant cells, but also result in significant acute and chronic toxicities in normal tissue and organs at risk (OAR). Interstitial pneumonitis is the major dose limiting toxicity for TBI, with lethal pulmonary toxicity correlating to the mean lung dose, driving a mean dose goal of 8 Gy.^1^

There has been a recent trend toward advanced techniques that take advantage of inverse planned modulated arc capabilities available with modern treatment planning systems. These techniques aim to spare OARs and healthy tissue, and selectively target malignant tissues. Further advancing the TBI technique led to total marrow irradiation (TMI) and total marrow and lymph node irradiation (TMLI) allowing selective targeting of the bone marrow and lymphoid tissue, and further sparing of normal and organ tissues. Helical tomotherapy (HT) based methods have been used for TMI/TMLI^2^, ^3^, ^4^, ^5^ as well as TBI.^5^, ^6^, ^7^ Using conventional linacs, intensity modulated radiotherapy (IMRT) TMI delivery proved feasible,^8^, ^9^ however, the predominant linac approach utilizes volumetric modulated arc therapy (VMAT) using Varian RapidArc (Varian Medical Systems, Palo Alto, CA, USA).^10^, ^11^, ^12^, ^13^, ^14^, ^15^, ^16^, ^17^

In October 2016, our institution began clinical VMAT TBI treatments on Elekta Agility linacs (Elekta Pty Ltd, Stockholm, Sweden), planned with the Pinnacle^3^ treatment planning system (TPS) (Philips Healthcare, Andover, MA, USA). 6 MV VMAT photon beams are delivered to four separate PTV sub‐volumes; head and neck, chest, abdomen, and pelvis (legs are treated with AP/PA beams). The PTV is defined as the entire body, contracted to 5 mm below the skin. A uniform dose of 12 Gy is prescribed to the PTV while limiting the mean lung dose to less than 8 Gy and mean kidney and liver doses to below 9 Gy. Based upon previously reported margins^3^, ^5^, ^13^, ^14^ the PTV was extended 3 mm into the lungs to account for setup, geometric, and intra‐fraction uncertainties. Patients are immobilized with a custom head and shoulder rest, thermoplastic mask, and full body vacuum bag. Imaging involves separate CBCT and shifts for each sub‐volume.

Over recent years, routine pre‐treatment quality assurance of IMRT and VMAT treatment plans has evolved beyond point dose measurements and film dosimetry to take advantage of modern ionization chamber or diode detector arrays.^18^, ^19^, ^20^, ^21^ Previous VMAT TBI and TMI/TMLI studies have utilized a variety of dose verification techniques. Portal imaging,^13^ Mapcheck,^14^ thermoluminescent dosimeters (TLDs) in an anthropomorphic phantom,^15^ ionization chamber array (Octavius)^16^ and diode array (ArcCHECK).^17^ Junction measurements are seldom reported on. Our institution has previously used film with an anthropomorphic phantom. Aydogan et al.^14^ reported on the measurement of the junction regions in VMAT TMI, using a pinpoint ionization chamber (0.0125 cm^3^) in a water phantom. Measurements were made at relatively low dose gradient points and evaluated using the differences in the measured and planned absolute dose measurements.

Our institution utilizes the ArcCHECK (Sun Nuclear Inc, Melbourne, FL, USA) diode array with a 3%/3 mm global gamma analysis (10% threshold) in SNC Patient version 6.6.2, with the ArcCHECK measurement uncertainty factor applied, for all routine VMAT QA. As such we have used the ArcCHECK array for dosimetric verification of all VMAT TBI beams, including junction measurements. The ArcCHECKs SNC Patient software can easily be used to measure a long field or two arcs with a shared isocentre, but requires some manipulation to measure a junction region between two VMAT arcs with separate isocentres. This study demonstrates a method to measure the dosimetry of a junction region between two adjacent VMAT TBI arcs from separate sub‐volumes (i.e., separate isocentres) with the ArcCHECK diode array.

## 2 MATERIALS AND METHODS

**Figure 1:**
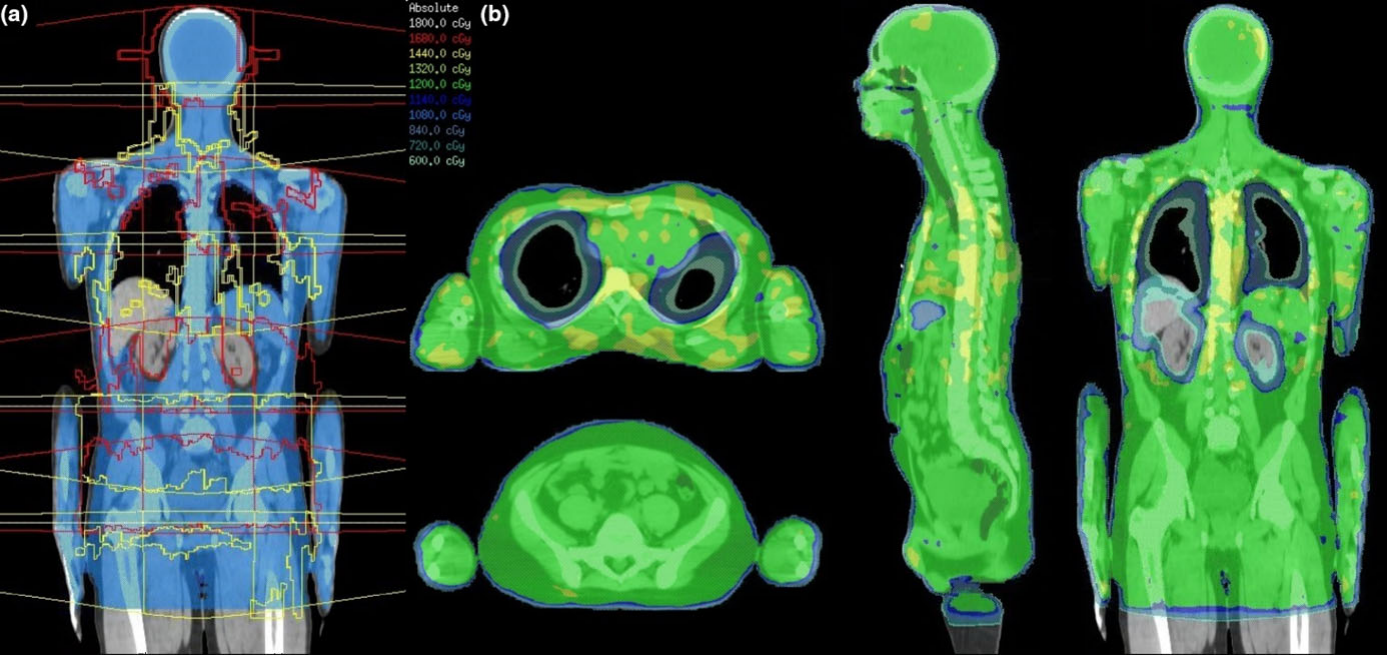
(a) Coronal view beam arrangement for each sub‐volume. The entire PTV (blue) is covered by eight arcs assigned to four isocentres, with each isocentre having a superior (red) and inferior (yellow) arc. (b) Final isodose colourwash after optimization of all arcs to deliver 12 Gy to the PTV, with sparing to the lungs, liver, and kidneys.

A total of 5 mm slice thickness CT images of our TBI patients are obtained from the top of the skull to the mid‐thigh. The PTV region (whole body, lungs contracted, skin contracted) is divided into sub‐volumes of the head, chest, abdomen, and pelvis. Each sub‐volume receives a centrally placed isocentre arranged along the patient's longitudinal axis with identical lateral and AP coordinates to limit couch movements during treatment to longitudinal shifts only [Fig. 1(a)]. Each isocentre receives two 360 degree VMAT arcs. As per previous planning studies, offset arcs with a 4 cm overlap and 90° collimator produced the best dose distribution.^11^, ^13^, ^14^ These are then optimized in stages on a 5 mm dose grid. Head and chest arcs are optimized and converted, then switched off as the abdominal and pelvic arcs are optimized and converted. All arcs are then switched on and optimized together, which aids in smoothing out junctions. The final dose calculation for all arcs is performed on a 3 mm dose grid.

Dosimetric verification of all VMAT plans (including TBI) at our centre is performed with the ArcCHECK cylindrical diode array. SNC's ArcCHECK is specifically designed for rotational dosimetry, comprised of an array of 1386 (0.016 mm^3^) diode detectors with a 1 cm spacing arranged in a helical pattern within a cylindrical PMMA phantom.^22^ Treatment plans are copied to an ArcCHECK phantom CT in the TPS, and calculated on a 2 mm dose grid.

**Figure 2:**
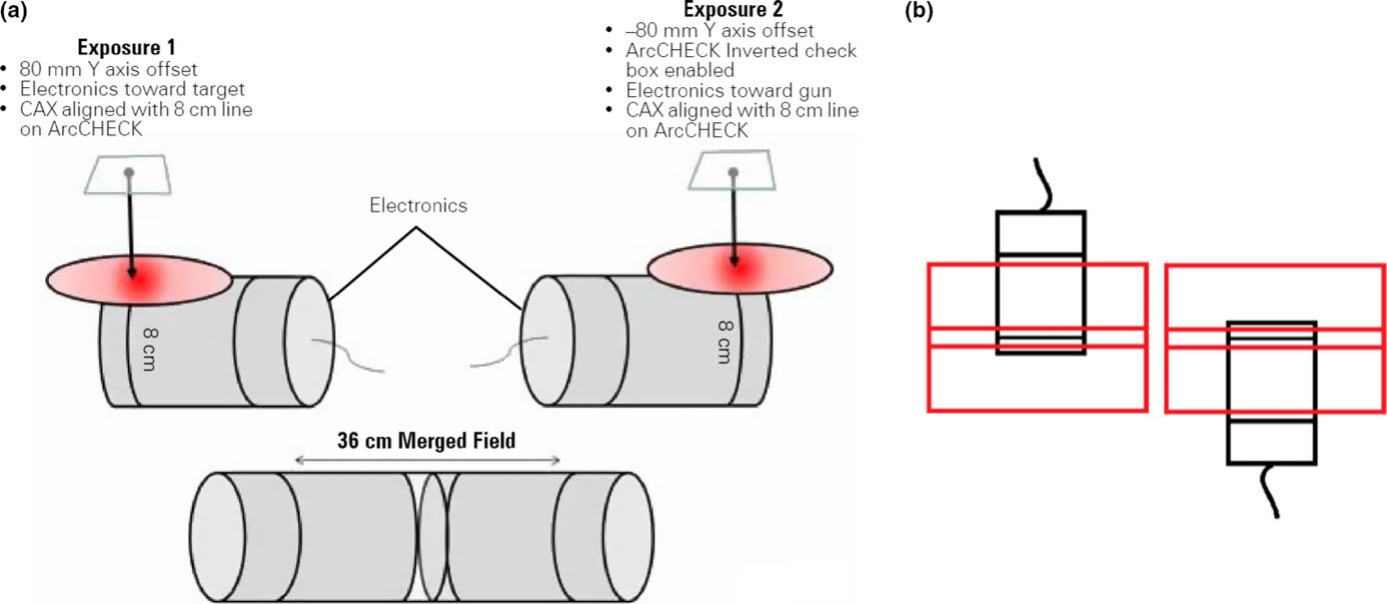
ArcCHECK inversion procedure for merging two exposures to accommodate extended field sizes (a), and schematic diagram of exposing two arcs from a single PTV sub‐volume (b).^23^

The ArcCHECKs normal operation allows for measurement of field sizes no greater than 20 cm, however, SNC Patient allows the merging of data from two exposures of extended field sizes up to 36 cm [Fig. 2(a)]. Two exposures of the same extended field are taken with the ArcCHECK in an inverted position. The software then stitches together the two exposures to create a single dose map. In the overlapping area between two exposures, the dose at each diode is averaged.

Although each VMAT TBI sub‐volume is comprised of two discrete arcs and not one large open field, the process is essentially the same as combined they are less than 36 cm. After exposure of both arcs from a sub‐volume (with one mostly in empty space), the ArcCHECK is inverted and both arcs are again delivered [Fig. 2(b)]. This process allows the creation of a measured dose map for the entire PTV sub‐volume. Note that the distance from the isocentre is limited 18 cm to avoid irradiation of the ArcCHECK's electronics.

**Figure 3:**
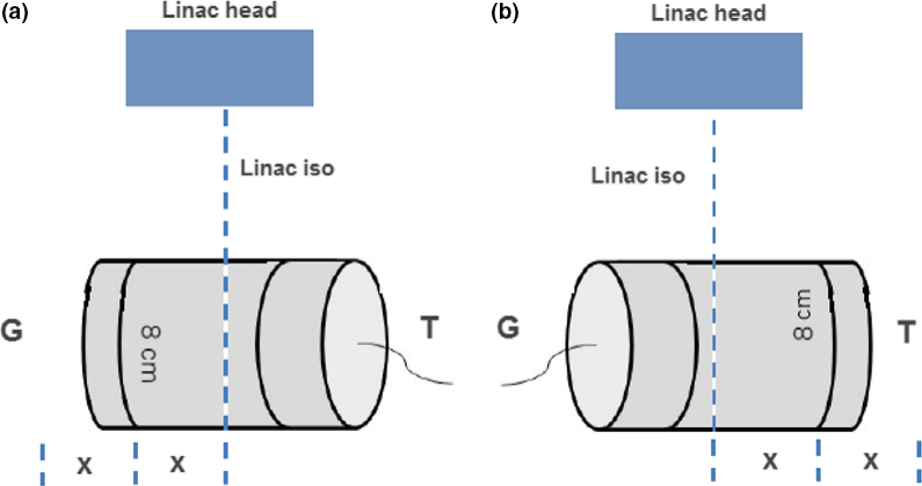
ArcCHECK setup method for measuring junction between two arcs from adjacent sub‐volumes, i.e., two overlapping arcs from separate isocentres.

Measuring of a junction region between two sub‐volumes offers some obstacles, namely that the central measurement area is not centered around an isocentre, but toward the field edges. In its normal operation, the isocentre of the beam to be measured (or shared isocentre of two beams) is placed at the center of the ArcCHECK phantom in the TPS. In the case of the junctions, the junction region needs to be placed at the center of the isocentre (Fig. 3). In Pinnacle, this requires the creation of a mock static beam and isocentre halfway between the isocentres of the junctioning beams that is then placed at the ArcCHECK center. The superior/inferior distance between the two isocentres, which is dictated by patient anatomy, is recorded as ‘2x’, with ‘x’ therefore being the distance to the central junction region (the isocentre of the mock static beam). Note that the distance ‘2x’ in Fig. 3 should not exceed 16 cm to ensure beams do not irradiate the phantom electronics.

The ArcCHECK is setup with the linac isocentre at the + 80 mm location, and the couch is then translated toward G by the distance ‘x’. The y‐coordinate offset in the SNC software is set to + 80 mm Y. The beam inferior to the junction is delivered, then without stopping the measurement in the SNC Patient software, the couch is translated toward T by a distance ‘2x’ and the beam superior to the junction is delivered. The SNC Patient software measurement is then stopped. The software effectively behaves as if it has just been delivered half of a single extended field arc. This process is repeated but with the ArcCHECK in the inverted position. The two measurements can then be merged in SNC patient to obtain a complete dose map of the junction region between two adjoining VMAT arcs.

## 3 RESULTS

**Figure 4:**
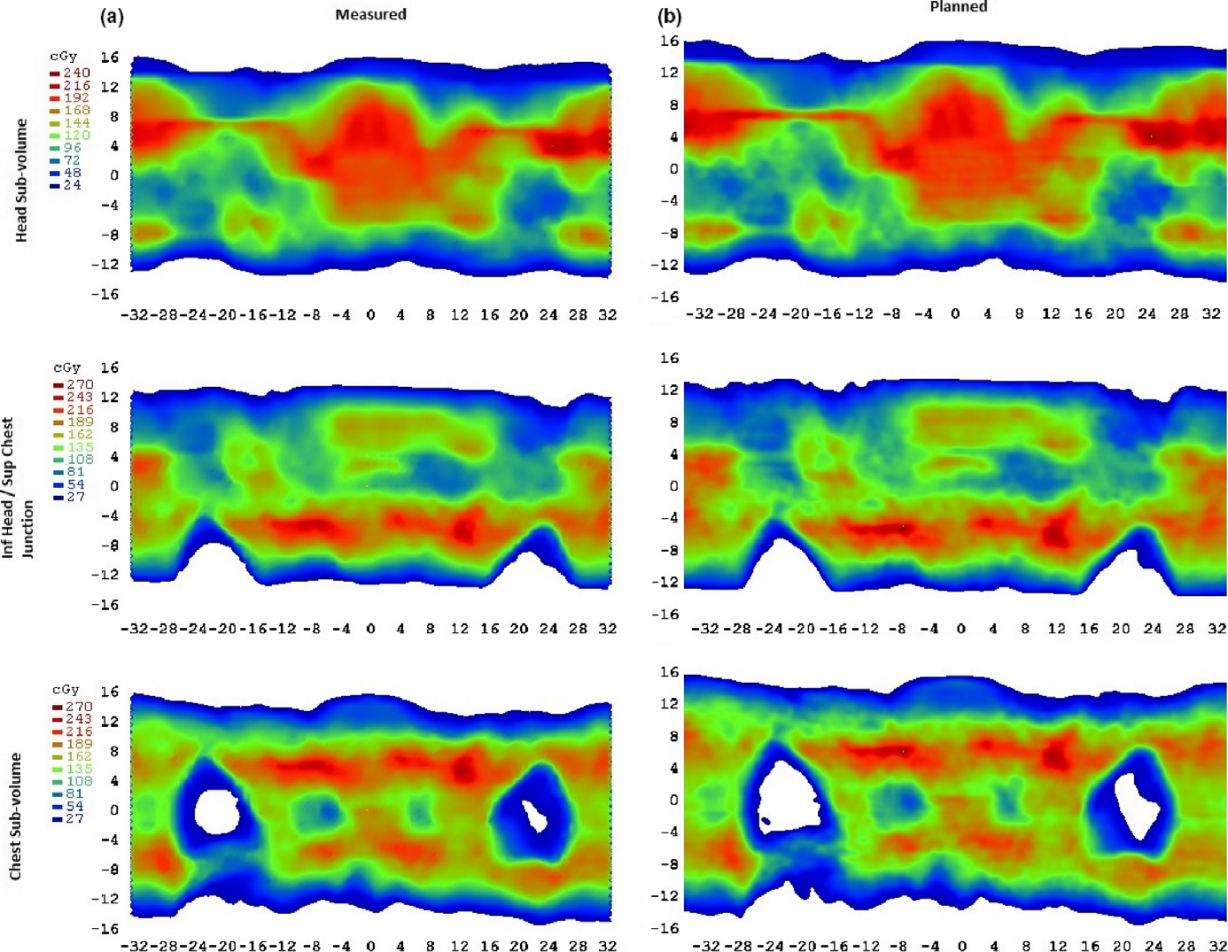
Single fraction colourwash isodose maps measured (a) and TPS planned (b), for a head sub‐volume (upper), a chest sub‐volume (lower), and the junction between the inferior head arc and superior chest arc (middle).

The VMAT TBI single fraction dose distribution analysis for a representative patient using SNC Patient 6.6.2 is shown in Fig. 4. Displayed are the measured and TPS planned dose distributions for the head sub‐volume, chest sub‐volume, and junction between these two. Measured and TPS dose distributions for each junction can be shown to closely match. Table 1 demonstrates agreement between the measured and planned dose distributions for all arcs for this patient, using a 3%/3 mm gamma analysis. Pass rates for arc junctions were found to be consistent with individual arc measurements, and all were greater than 97.5%, above the 95% tolerance required for normal clinical VMAT arcs at our institution.

**Table 1:** 3%/3 mm absolute dose gamma analysis results for all arcs for the representative patient

PTV	Beam	% Pass
Head	Head 1	99.4
	Head 2	99.2
	Head 1 & 2	99.7
	Head 2 & Chest 1	99.9
Chest	Chest 1	100
	Chest 2	99.8
	Chest 1 & 2	98.7
	Chest 2 & Abdo 1	98.3
Abdo	Abdo 1	98.4
	Abdo 2	99.7
	Abdo 1 & 2	97.5
	Abdo 2 & Pelv 1	100
Pelvic	Pelv 1	99.8
	Pelv 2	100
	Pelv 1 & 2	100

## 4 DISCUSSION

The Sun Nuclear ArcCHECK diode array routinely used for VMAT QA was utilized for measurement of junction regions between adjacent VMAT TBI arcs. This allows for a comprehensive analysis of junction regions with a diode array, superseding the need to use film or point dose measurements. It must be noted, however, that fields and their junctions require more comprehensive measurements during the commissioning phase of a VMAT TBI technique. In addition to ArcCHECK measurements, our institution utilized an anthropomorphic phantom with internal hole grids that allowed internal dosimetry to be collected with 180 TLDs. The segmented assembly of the phantom also allowed transverse films to be taken, and additionally had cranial and rectal ion chamber cavities for additional point dose measurements. Film measurements in solid water within the coronal plane were also taken. A thorax phantom allowed point dose ion chamber and transverse film analysis in lung equivalent tissue.

While the 3%/3 mm gamma analysis metric is routinely used in the clinical environment for pre‐treatment QA of VMAT and IMRT plans, it is worth noting studies such as those by Fredh et al.,^24^ Heilemann et al.,^25^ and Nelms et al.^26^ that discuss implementing stricter gamma tolerances based on concerns of sensitivity in detecting introduced errors. Although the particulars of gamma analysis fall outside the scope of this study, interested readers are directed to the aforementioned references for more information.

**Figure 5:**
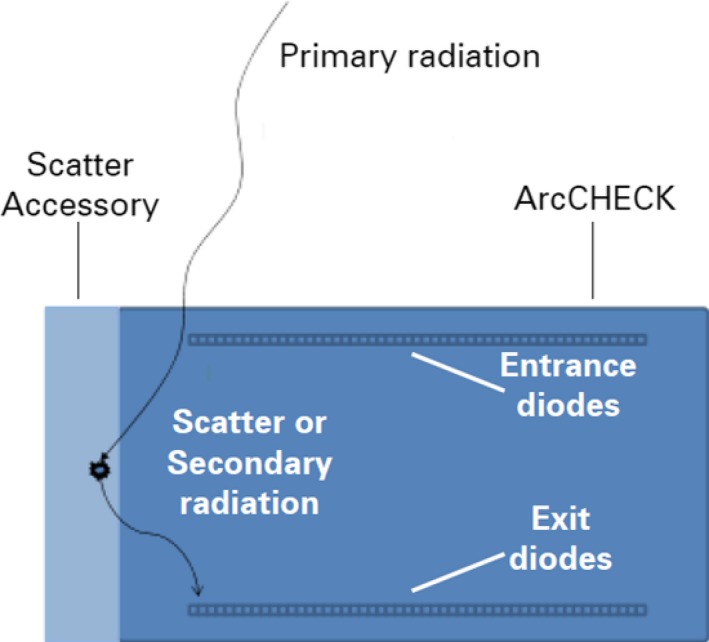
Cross‐section of the ArcCHECK phantom with attached scatter accessory demonstrating scatter and secondary radiation originating from within the scatter accessory and contributing to exit diode signals.^23^

There is a considerable time factor involved with a full QA of all individual arcs, sub‐volume junctions, and inter sub‐volume junctions, requiring multiple deliveries of each arc with alternate phantom placements and couch shifts. The time for physics QA is approximately 6 h for dose calculation on the QA phantom, and 2–3 h of machine measurements. Once confidence in junction regions has been established during the commissioning of a VMAT TBI technique, QA may only need to be performed on individual arcs for successive patients, providing a robust MLC QA is performed regularly (as junctions between PTVs would be at the edges of MLC travel and errors in this area may be difficult to pick up with the ArcCHECK measurements alone). Analysis could become more robust if there was further consideration spent on scatter and secondary radiation when measuring fields that extend beyond the edge of the phantom. A scatter accessory is available for the ArcCHECK phantom that introduces an extra 9.1 cm of high density polyethylene for long field measurements (Fig. 5), however, our institution has yet to investigate its impact on QA pass rates for such fields.^23^

This technique is of course not limited to TBI, it can be applied to any treatment modality in which junctions exist between VMAT fields, and has been utilized at our institution for VMAT CNS treatments.

## 5 CONCLUSION

This study demonstrates how to use the commercially available ArcCHECK phantom and software for dosimetric analysis of the junction region between two VMAT arcs with separate isocentres in the context of VMAT TBI. This technique requires no extra equipment beyond what is already used for routine QA, and provides dosimetric confidence for the junction regions of overlapping arcs.

## CONFLICT OF INTEREST

The authors declare no conflict of interest.
